# *tert*-Butyl 2-(1*H*-benzimidazol-1-yl)acetate

**DOI:** 10.1107/S1600536812002814

**Published:** 2012-02-04

**Authors:** Nassir N. Al-Mohammed, Yatimah Alias, Zanariah Abdullah, Hamid Khaledi

**Affiliations:** a Department of Chemistry, University of Malaya, 50603 Kuala Lumpur, Malaysia

## Abstract

In the title compound, C_13_H_16_N_2_O_2_, the planes of the benzimidazole ring system and the acetate O—C=O fragment make a dihedral angle of 84.5 (3)°. In the crystal, mol­ecules are connected through C—H⋯N hydrogen bonds to form infinite chains in the [-110] direction.

## Related literature

For related structures, see: Al-Mohammed *et al.* (2012 ▶); Fu *et al.* (2009 ▶); Xu *et al.* (2008 ▶).
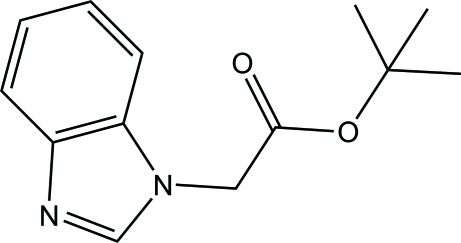


## Experimental

### 

#### Crystal data

C_13_H_16_N_2_O_2_
*M* *_r_* = 232.28
Monoclinic, 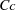

*a* = 5.4204 (2) Å
*b* = 11.3319 (4) Å
*c* = 19.8771 (7) Å
β = 96.609 (3)°
*V* = 1212.81 (8) Å^3^
*Z* = 4
Mo *K*α radiation
μ = 0.09 mm^−1^
*T* = 100 K
0.19 × 0.13 × 0.04 mm

#### Data collection

Bruker APEXII CCD diffractometer
Absorption correction: multi-scan (*SADABS*; Sheldrick, 1996 ▶) *T*_min_ = 0.984, *T*_max_ = 0.997
3829 measured reflections
1244 independent reflections
1091 reflections with *I* > 2σ(*I*)
*R*_int_ = 0.036

#### Refinement

*R*[*F* ^2^ > 2σ(*F* ^2^)] = 0.034
*wR*(*F* ^2^) = 0.076
*S* = 1.04
1244 reflections
157 parameters
2 restraints
H-atom parameters constrained
Δρ_max_ = 0.17 e Å^−3^
Δρ_min_ = −0.20 e Å^−3^

### 

Data collection: *APEX2* (Bruker, 2007 ▶); cell refinement: *APEX2*; data reduction: *SAINT* (Bruker, 2007 ▶); program(s) used to solve structure: *SHELXS97* (Sheldrick, 2008 ▶); program(s) used to refine structure: *SHELXL97* (Sheldrick, 2008 ▶); molecular graphics: *X-SEED* (Barbour, 2001 ▶); software used to prepare material for publication: *SHELXL97* and *publCIF* (Westrip, 2010 ▶).

## Supplementary Material

Crystal structure: contains datablock(s) I, global. DOI: 10.1107/S1600536812002814/gk2453sup1.cif

Structure factors: contains datablock(s) I. DOI: 10.1107/S1600536812002814/gk2453Isup2.hkl

Supplementary material file. DOI: 10.1107/S1600536812002814/gk2453Isup3.cml

Additional supplementary materials:  crystallographic information; 3D view; checkCIF report

## Figures and Tables

**Table 1:** Hydrogen-bond geometry (Å, °)

*D*—H⋯*A*	*D*—H	H⋯*A*	*D*⋯*A*	*D*—H⋯*A*
C3—H3⋯N2^i^	0.95	2.48	3.406 (3)	165

Symmetry code: (i) 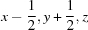.

**Fig. 1.**
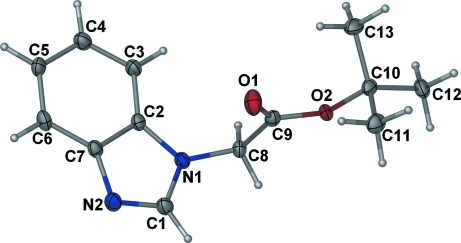
The molecular structure of the title compound showing displacement ellipsoids at the 50% probability level. Hydrogen atoms are drawn as spheres of arbitrary radius.

## supplementary crystallographic information

### Comment

The title compound is the condensation product of the reaction of benzimidazole with *tert*-butyl chloroacetate. The benzimidazole ring make a dihedral angle of 84.5 (3)° with the plane passing through the acetate group, C9/O1/O2. This value is comparable to those calculated for some similar structures (Al-Mohammed *et al.*, 2012; Fu *et al.*, 2009; Xu *et al.*, 2008). The crystal packing structure contains chains in [-1 1 0] direction formed by C3—H3···N2 hydrogen bonds between the adjacent molecules. Intramolecular C—H···O hydrogen bonding also occurs.

### Experimental

Sodium hydroxide (0.5 g, 125 mmol) was added to a solution of benzimidazole (1.48 g, 125 mmol) in DMF (20 ml), followed by addition of *tert*-butyl chloroacetate (1.82 ml, 127 mmol). The mixture was refluxed for 1 h. The reaction mass was quenched with cold water (50 ml) and extracted by dichloromethane (3 *x* 25 ml). The combined organic layers was washed with cold water and brine and dried over anhydrous sodium sulfate. The solvent was evaporated under vacuum and the formed amorphous solid was stirred in *n*-hexane (30 ml) at room temperature. The solid was filtered, washed with hexane (2 *x* 20 ml), and recrystallized from ethyl acetate to afford colorless crystals of the title compound (melting point = 367–369 K).

### Refinement

Hydrogen atoms were placed at calculated positions and refined in riding mode with C—H distances of 0.95 (aromatic), 0.98 (methyl) and 0.99 (methylene) Å, and *U*_iso_(H) set to 1.2 (1.5 for methyl) *U*_eq_(carrier atoms). In the absence of significant anomalous scattering effects, Friedel pairs have been merged.

### Crystal data

**Table:**

C_13_H_16_N_2_O_2_	*F*(000) = 496
*M**_r_* = 232.28	*D*_x_ = 1.272 Mg m^−^^3^
Monoclinic, *C**c*	Mo *K*α radiation, λ = 0.71073 Å
Hall symbol: C -2yc	Cell parameters from 1057 reflections
*a* = 5.4204 (2) Å	θ = 3.6–26.4°
*b* = 11.3319 (4) Å	µ = 0.09 mm^−^^1^
*c* = 19.8771 (7) Å	*T* = 100 K
β = 96.609 (3)°	Tablet, colourless
*V* = 1212.81 (8) Å^3^	0.19 × 0.13 × 0.04 mm
*Z* = 4	

### Data collection

**Table:**

Bruker APEXII CCD diffractometer	1244 independent reflections
Radiation source: fine-focus sealed tube	1091 reflections with *I* > 2σ(*I*)
Graphite monochromator	*R*_int_ = 0.036
φ and ω scans	θ_max_ = 26.5°, θ_min_ = 3.6°
Absorption correction: multi-scan (*SADABS*; Sheldrick, 1996)	*h* = −6→6
*T*_min_ = 0.984, *T*_max_ = 0.997	*k* = −14→14
3829 measured reflections	*l* = −24→24

### Refinement

**Table:**

Refinement on *F*^2^	Primary atom site location: structure-invariant direct methods
Least-squares matrix: full	Secondary atom site location: difference Fourier map
*R*[*F*^2^ > 2σ(*F*^2^)] = 0.034	Hydrogen site location: inferred from neighbouring sites
*wR*(*F*^2^) = 0.076	H-atom parameters constrained
*S* = 1.04	*w* = 1/[σ^2^(*F*_o_^2^) + (0.0394*P*)^2^ + 0.180*P*] where *P* = (*F*_o_^2^ + 2*F*_c_^2^)/3
1244 reflections	(Δ/σ)_max_ < 0.001
157 parameters	Δρ_max_ = 0.17 e Å^−^^3^
2 restraints	Δρ_min_ = −0.20 e Å^−^^3^

### Special details

**Table:**

Geometry. All e.s.d.'s (except the e.s.d. in the dihedral angle between two l.s. planes) are estimated using the full covariance matrix. The cell e.s.d.'s are taken into account individually in the estimation of e.s.d.'s in distances, angles and torsion angles; correlations between e.s.d.'s in cell parameters are only used when they are defined by crystal symmetry. An approximate (isotropic) treatment of cell e.s.d.'s is used for estimating e.s.d.'s involving l.s. planes.
Refinement. Refinement of *F*^2^ against ALL reflections. The weighted *R*-factor *wR* and goodness of fit *S* are based on *F*^2^, conventional *R*-factors *R* are based on *F*, with *F* set to zero for negative *F*^2^. The threshold expression of *F*^2^ > σ(*F*^2^) is used only for calculating *R*-factors(gt) *etc*. and is not relevant to the choice of reflections for refinement. *R*-factors based on *F*^2^ are statistically about twice as large as those based on *F*, and *R*- factors based on ALL data will be even larger.

### Fractional atomic coordinates and isotropic or equivalent isotropic displacement parameters (Å^2^)

**Table:**

	*x*	*y*	*z*	*U*_iso_*/*U*_eq_	
O1	0.0330 (4)	0.41863 (16)	0.04890 (10)	0.0302 (5)	
O2	0.2082 (3)	0.59850 (13)	0.07085 (9)	0.0174 (4)	
N1	0.3020 (3)	0.38525 (17)	−0.05825 (10)	0.0193 (5)	
N2	0.3571 (4)	0.19170 (18)	−0.07541 (12)	0.0243 (5)	
C1	0.4419 (5)	0.2870 (2)	−0.04255 (14)	0.0227 (6)	
H1	0.5871	0.2872	−0.0108	0.027*	
C2	0.1057 (4)	0.3507 (2)	−0.10483 (12)	0.0183 (5)	
C3	−0.0961 (4)	0.4127 (2)	−0.13641 (13)	0.0222 (6)	
H3	−0.1195	0.4943	−0.1282	0.027*	
C4	−0.2609 (5)	0.3489 (2)	−0.18058 (14)	0.0250 (6)	
H4	−0.4014	0.3879	−0.2035	0.030*	
C5	−0.2268 (5)	0.2281 (2)	−0.19258 (13)	0.0228 (6)	
H5	−0.3446	0.1872	−0.2232	0.027*	
C6	−0.0250 (5)	0.1677 (2)	−0.16060 (14)	0.0225 (6)	
H6	−0.0019	0.0862	−0.1690	0.027*	
C7	0.1446 (4)	0.2301 (2)	−0.11554 (13)	0.0192 (5)	
C8	0.3234 (4)	0.4973 (2)	−0.02268 (13)	0.0183 (5)	
H8A	0.2671	0.5617	−0.0544	0.022*	
H8B	0.4997	0.5119	−0.0056	0.022*	
C9	0.1684 (4)	0.4977 (2)	0.03638 (13)	0.0187 (5)	
C10	0.0928 (4)	0.6199 (2)	0.13424 (13)	0.0200 (5)	
C11	0.1856 (5)	0.5282 (2)	0.18666 (14)	0.0264 (6)	
H11A	0.1199	0.4505	0.1722	0.040*	
H11B	0.1293	0.5487	0.2303	0.040*	
H11C	0.3675	0.5260	0.1914	0.040*	
C12	0.1849 (5)	0.7429 (2)	0.15475 (15)	0.0256 (6)	
H12A	0.3668	0.7432	0.1612	0.038*	
H12B	0.1214	0.7655	0.1971	0.038*	
H12C	0.1257	0.7992	0.1191	0.038*	
C13	−0.1878 (5)	0.6198 (2)	0.11925 (15)	0.0271 (6)	
H13A	−0.2380	0.6743	0.0819	0.041*	
H13B	−0.2616	0.6451	0.1596	0.041*	
H13C	−0.2454	0.5400	0.1065	0.041*	

### Atomic displacement parameters (Å^2^)

**Table:**

	*U*^11^	*U*^22^	*U*^33^	*U*^12^	*U*^13^	*U*^23^
O1	0.0402 (11)	0.0241 (10)	0.0279 (11)	−0.0125 (8)	0.0109 (9)	−0.0047 (9)
O2	0.0196 (9)	0.0158 (8)	0.0172 (9)	0.0005 (7)	0.0035 (7)	−0.0026 (7)
N1	0.0221 (11)	0.0165 (10)	0.0191 (12)	0.0029 (8)	0.0013 (9)	−0.0011 (9)
N2	0.0288 (12)	0.0191 (11)	0.0239 (12)	0.0041 (9)	−0.0013 (10)	−0.0012 (10)
C1	0.0253 (14)	0.0225 (13)	0.0196 (13)	0.0059 (11)	0.0001 (11)	−0.0002 (11)
C2	0.0214 (13)	0.0185 (12)	0.0153 (14)	0.0003 (10)	0.0037 (10)	−0.0018 (10)
C3	0.0249 (14)	0.0187 (12)	0.0224 (14)	0.0045 (10)	0.0001 (11)	−0.0023 (11)
C4	0.0273 (14)	0.0250 (14)	0.0221 (15)	0.0067 (11)	0.0008 (12)	−0.0008 (11)
C5	0.0266 (14)	0.0251 (14)	0.0163 (13)	0.0001 (11)	−0.0001 (11)	−0.0051 (11)
C6	0.0317 (15)	0.0164 (12)	0.0197 (14)	0.0010 (10)	0.0045 (11)	−0.0024 (10)
C7	0.0242 (14)	0.0186 (12)	0.0148 (13)	0.0031 (10)	0.0022 (10)	−0.0004 (10)
C8	0.0205 (12)	0.0176 (12)	0.0167 (13)	−0.0013 (10)	0.0019 (10)	−0.0030 (10)
C9	0.0210 (12)	0.0180 (12)	0.0163 (13)	0.0017 (10)	−0.0011 (10)	0.0007 (10)
C10	0.0188 (13)	0.0264 (13)	0.0152 (13)	−0.0015 (10)	0.0036 (10)	−0.0033 (11)
C11	0.0268 (14)	0.0341 (14)	0.0180 (13)	−0.0007 (12)	0.0016 (11)	0.0008 (12)
C12	0.0255 (13)	0.0250 (12)	0.0273 (15)	−0.0010 (11)	0.0071 (11)	−0.0092 (12)
C13	0.0167 (13)	0.0394 (15)	0.0255 (15)	−0.0004 (11)	0.0031 (11)	−0.0029 (13)

### Geometric parameters (Å, º)

**Table:**

O1—C9	1.203 (3)	C6—C7	1.399 (4)
O2—C9	1.336 (3)	C6—H6	0.9500
O2—C10	1.490 (3)	C8—C9	1.520 (3)
N1—C1	1.363 (3)	C8—H8A	0.9900
N1—C2	1.384 (3)	C8—H8B	0.9900
N1—C8	1.451 (3)	C10—C11	1.516 (4)
N2—C1	1.317 (3)	C10—C13	1.516 (3)
N2—C7	1.394 (3)	C10—C12	1.520 (4)
C1—H1	0.9500	C11—H11A	0.9800
C2—C3	1.388 (3)	C11—H11B	0.9800
C2—C7	1.402 (3)	C11—H11C	0.9800
C3—C4	1.383 (4)	C12—H12A	0.9800
C3—H3	0.9500	C12—H12B	0.9800
C4—C5	1.406 (4)	C12—H12C	0.9800
C4—H4	0.9500	C13—H13A	0.9800
C5—C6	1.382 (4)	C13—H13B	0.9800
C5—H5	0.9500	C13—H13C	0.9800
			
C9—O2—C10	120.91 (18)	C9—C8—H8B	109.4
C1—N1—C2	106.6 (2)	H8A—C8—H8B	108.0
C1—N1—C8	126.3 (2)	O1—C9—O2	126.7 (2)
C2—N1—C8	125.84 (19)	O1—C9—C8	124.2 (2)
C1—N2—C7	104.24 (19)	O2—C9—C8	109.14 (18)
N2—C1—N1	113.8 (2)	O2—C10—C11	109.36 (19)
N2—C1—H1	123.1	O2—C10—C13	110.1 (2)
N1—C1—H1	123.1	C11—C10—C13	112.4 (2)
N1—C2—C3	131.6 (2)	O2—C10—C12	102.64 (19)
N1—C2—C7	105.1 (2)	C11—C10—C12	111.8 (2)
C3—C2—C7	123.3 (2)	C13—C10—C12	110.1 (2)
C4—C3—C2	116.1 (2)	C10—C11—H11A	109.5
C4—C3—H3	121.9	C10—C11—H11B	109.5
C2—C3—H3	121.9	H11A—C11—H11B	109.5
C3—C4—C5	122.0 (2)	C10—C11—H11C	109.5
C3—C4—H4	119.0	H11A—C11—H11C	109.5
C5—C4—H4	119.0	H11B—C11—H11C	109.5
C6—C5—C4	121.1 (2)	C10—C12—H12A	109.5
C6—C5—H5	119.5	C10—C12—H12B	109.5
C4—C5—H5	119.5	H12A—C12—H12B	109.5
C5—C6—C7	118.2 (2)	C10—C12—H12C	109.5
C5—C6—H6	120.9	H12A—C12—H12C	109.5
C7—C6—H6	120.9	H12B—C12—H12C	109.5
N2—C7—C6	130.4 (2)	C10—C13—H13A	109.5
N2—C7—C2	110.3 (2)	C10—C13—H13B	109.5
C6—C7—C2	119.4 (2)	H13A—C13—H13B	109.5
N1—C8—C9	111.03 (18)	C10—C13—H13C	109.5
N1—C8—H8A	109.4	H13A—C13—H13C	109.5
C9—C8—H8A	109.4	H13B—C13—H13C	109.5
N1—C8—H8B	109.4		

### Hydrogen-bond geometry (Å, º)

**Table:**

*D*—H···*A*	*D*—H	H···*A*	*D*···*A*	*D*—H···*A*
C11—H11*A*···O1	0.98	2.47	3.033 (3)	116
C13—H13*C*···O1	0.98	2.42	2.995 (3)	117
C3—H3···N2^i^	0.95	2.48	3.406 (3)	165

Symmetry code: (i) *x*−1/2, *y*+1/2, *z*.
